# Correction: Krivonogov et al. Microbial Diversity and Authigenic Mineral Formation of Modern Bottom Sediments in the Littoral Zone of Lake Issyk-Kul, Kyrgyz Republic (Central Asia). *Biology* 2023, *12*, 642

**DOI:** 10.3390/biology12081109

**Published:** 2023-08-09

**Authors:** Sergei Krivonogov, Anton Maltsev, Darya Zelenina, Alexey Safonov

**Affiliations:** 1 Faculty of Geosciences and Environmental Engineering, Southwest Jiaotong University, Chengdu 611756, China; carpos.sergey@gmail.com; 2 V.S. Sobolev Institute of Geology and Mineralogy Siberian Branch, RAS, Novosibirsk 630090, Russia; 3 A.N. Frumkin Institute of Physical Chemistry and Electrochemistry, Russian Academy of Sciences, Moscow 119071, Russia; den-taranee.92@mail.ru

## 1. Addition of an Author

Sergei Krivonogov was not included as an author in the original publication [1]. The corrected Author Contributions statement appears here.

**Author Contributions:** Conceptualization S.K., A.S. and A.M.; methodology, A.M. and A.S.; software, D.Z.; validation, D.Z., A.M. and A.S.; formal analysis, A.S.; investigation, A.M. and D.Z.; resources, S.K. and A.M.; data curation, D.Z.; writing—original draft preparation, D.Z., A.S. and A.M.; writing—review and editing, S.K., D.Z. and A.S.; visualization, D.Z. and A.M.; supervision, S.K. and A.S.; project administration, S.K.; funding acquisition, S.K. All authors have read and agreed to the published version of the manuscript.

## 2. Additional Affiliation

In the published publication [1], there was an error regarding the affiliation for Sergei Krivonogov. In addition to affiliation 1, the updated affiliation should include: Faculty of Geosciences and Environmental Engineering, Southwest Jiaotong University, Chengdu 611756, China. 

## 3. Correction Funding and Acknowledgments

In the original publication [1], the funder “V.S. Sobolev Institute of Geology and Mineralogy Siberian Branch Russian Academy of Sciences” and “The project N 122041400193-7.” were not included. A correction has been made to the “Funding” and “Acknowledgments” sections:

**Funding:** This research was funded by RFBR-NSFC grant 21-55-53037 and state assignments from The Ministry of Science and Higher Education of the Russian Federation (#AAAA-A16-11611091001 and 0330-2016-0011) and performed using the equipment of the Core Facilities Center of IPCE RAS (CKP FMI IPCE RAS) and the Analytical Center for Multi-element and Isotope Studies of the V.S. Sobolev Institute of Geology and Mineralogy (Novosibirsk). Work was done on state assignment of V.S. Sobolev Institute of Geology and Mineralogy Siberian Branch Russian Academy of Sciences, Project N 122041400193-7.

We changed the acknowledgements accordingly:

**Acknowledgments:** The authors are grateful to Rysbek Satylkanov, of the Tien Shan High Mountain Scientific Centre National Academy of Sciences of the Kyrgyz Republic, for hosting our field work. We thank Nadezhda Popova and Denis Sobolev for their laboratory assistance.

The authors apologize for any inconvenience caused and state that the scientific conclusions are unaffected. This correction was approved by the Academic Editor. The original publication has also been updated.
