# Supplementary material for: Microbial Diversity and Authigenic Mineral Formation of Modern Bottom Sediments in the Littoral Zone of Lake Issyk-Kul, Kyrgyz Republic (Central Asia)
Source: Biology (Basel). 2023 Apr 23;12(5):642. doi: 10.3390/biology12050642 (PMC10215221; doi:10.3390/biology12050642)
Supplement: Supplementary file 1 [file biology-12-00642-s001.zip › biology-2232983-supplementary.pdf]

Supplimentary Materials

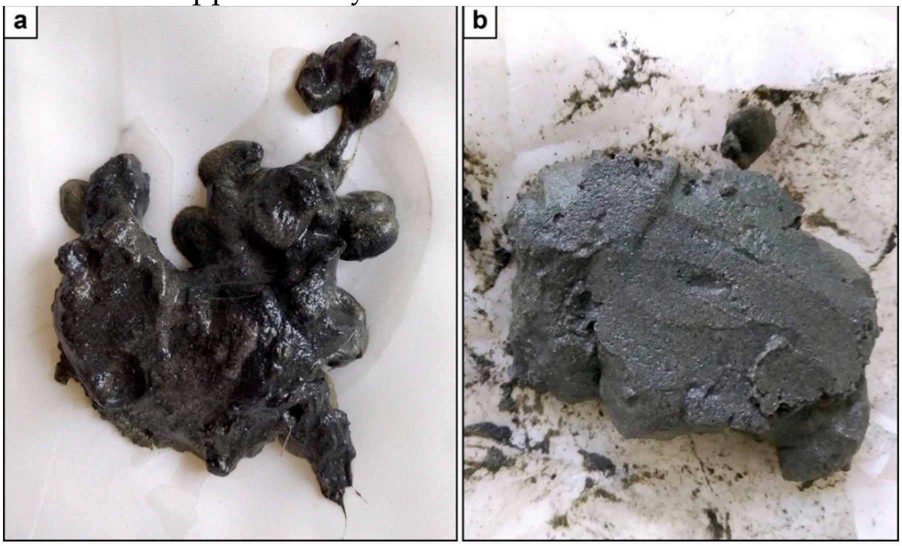

Figure S1. Bottom sediment samples from point IS-1. a. black silt in 0–5 cm; b. gray silt in 25–30 cm.

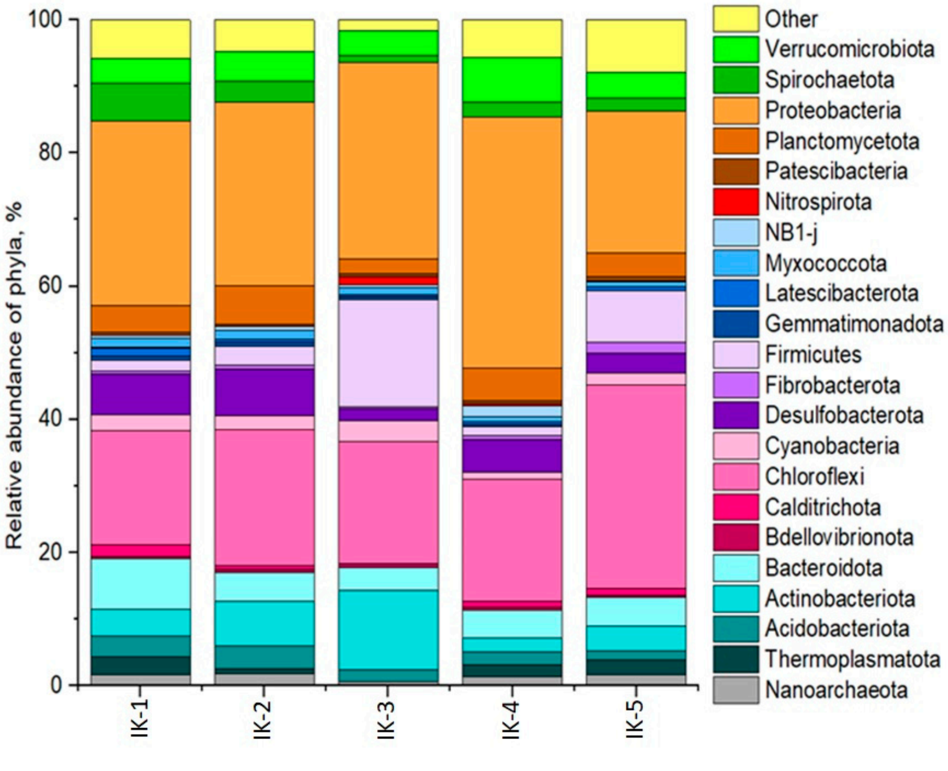

Figure S2. Phylogenetic diversity of bottom sediment microbial communities based on the analysis of 16S rRNA gene sequences at the phylum level.

Table S1. Diversity index of nicrobial communities in bottom sediments samples.

Sample	IK-1	IK-2	IK-3	IK-4	IK-5
OTU	422	463	394	395	503
Simpson_1-D	0.9795	0.981	4.902	0.9453	0.9594
Shannon_H	4.856	5.008	0.3416	4.247	4.51
Equitability_J	0.8034	0.8159	0.8203	0.7103	0.725
